# Corporate Advertising

**Published:** 1908-12-12

**Authors:** Geo. A. Arnaudin

**Affiliations:** Secretary, St. John's Hospital for Diseases of the Skin, Leicester Square, London, W.C.


### EDITOR'S LETTER-BOX.

**Our correspondents are reminded that prolixity is a great bar to publication, and that brevity of style and conciseness of statement greatly facilitate early insertion.**

### "CORPORATE ADVERTISING."

*To the Editor of* THE HOSPITAL.

SIR,—With reference to your "Annotation" which appeared in THE HOSPITAL of the 5th inst. condemning the form of advertisement recently issued on behalf of this hospital, I write at once to say that the medical staff, either collectively or individually, had absolutely nothing to do with the wording or issue of the advertisement.

I quite agree with your concluding paragraph, "A sense of solidarity is an excellent thing, except when allowed to override all scruple and delicacy." I do *not* agree that this is a just comment upon the advertisement referred to; but as it appears to have attracted censure when it was intended only to emphasise the valuable work being done in a small special hospital, I have taken steps for its immediate withdrawal.

I beg to remain, your obedient servant,

GEO. A. ARNAUDIN,

Secretary.

St. John's Hospital for Diseases of the Skin,
Leicester Square, London, W.C.,
December 7, 1908.

[We are glad to hear that the advertisement in question is withdrawn.—ED. T. H.]

